# The Inhibitory Effects of 6-Thioguanine and 6-Mercaptopurine on the USP2a Target Fatty Acid Synthase in Human Submaxillary Carcinoma Cells

**DOI:** 10.3389/fonc.2021.749661

**Published:** 2021-12-10

**Authors:** Chiao-Pei Cheng, Shu-Ting Liu, Yi-Lin Chiu, Shih-Ming Huang, Ching-Liang Ho

**Affiliations:** ^1^ Department of Anesthesiology, Tri-Service General Hospital, National Defense Medical Center, Taipei City, Taiwan; ^2^ Department of Biochemistry, National Defense Medical Center, Taipei City, Taiwan; ^3^ Division of Hematology and Oncology, Department of Medicine, Tri-Service General Hospital, National Defense Medical Center, Taipei City, Taiwan

**Keywords:** ubiquitin, USP2a, fatty acid synthase, cyclin D1, thiopurine

## Abstract

Overexpression of the deubiquitinase USP2a leads to stabilization of fatty acid synthase (FAS), the levels of which are often elevated in aggressive human cancers. Consequently, there is an urgent need for inhibitors to suppress the deubiquitination activity of USP2a so as to upregulate FAS protein degradation. We first analyzed the relationship between the expression level of USP2a and survival using The Cancer Genome Atlas Head-Neck Squamous Cell Carcinoma (HNSC) data collection. Our results suggested survival rates were lower among HNSC patients expressing higher levels of USP2a. We then investigated two thiopurine drugs, 6-thioguanine (6-TG) and 6-mercaptopurine (6-MP), to determine whether they could potentially serve as inhibitors of USP2a. Western blot analysis showed that levels of two USP2a target proteins, FAS and Mdm2, were dose-dependently decreased in A253 submaxillary carcinoma cells treated with 6-TG or 6‐MP. Responding to the degradation of Mdm2, levels of p53 were increased. We found that 6-TG and 6-MP also suppressed levels of both USP2a mRNA and protein, suggesting these two thiopurines do not act solely through direct inhibition of USP2a. The effects of 6-TG and 6-MP were not cell type-specific, as they elicited similar decreases in FAS protein in leukemia, prostate and cervical cancer cell lines. 6-TG and 6-MP had effects on several cell cycle proteins, including another USP2a target protein, cyclin D1. The populations of cells in subG1 and S phase were increased by 6-TG and 6-MP, which was accompanied by reductions in G1 phase cells. In untreated cells, USP2a transfection increased FAS and cyclin D1 levels compared to an enzyme-dead USP2a C276A mutant, which lacked deubiquitinating activity. However, USP2a transfection failed to reverse the suppressive effects of 6‐TG and 6-MP on FAS levels. In summary, these findings suggest 6-TG and 6-MP reduce the stability of some USP2a targets, including FAS and Mdm2, by inhibiting USP2a-catalyzed deubiquitination in some cancer cells. Our work also provides repurposing evidence supporting 6‐TG and 6-MP as target therapeutic drugs, such as USP2a/FAS in this study.

## Introduction

By mediating the degradation of short-lived and abnormal proteins, ubiquitination plays critical roles in the growth, environmental adaptation, development, and stress responses of eukaryotic cells. Indeed, the ubiquitin/26S proteasome proteolytic pathway plays important roles in virtually all aspects of cell biology (1, 2). Reversal of ubiquitination is accomplished through deubiquitinases (DUBs) (3, 4). Within the DUB family, ubiquitin-specific proteases (USPs) constitute the largest subgroup, with more than 60 members. USPs may help regulate the ubiquitin-dependent 26S proteasome degradation pathway by generating free ubiquitin monomers, recycling ubiquitin, and/or removing ubiquitin from specific targets, thereby preventing target degradation. Moreover, increasing evidence now indicates that alterations in DUB expression frequency alterations as well as DUB gene mutations correlate with human diseases, ranging from immune system diseases to human cancers.

USP2a is an androgen-regulated DUB reportedly overexpressed in prostate tumors, where it exerts an anti-apoptotic effect (5–7). In addition, in biologically aggressive human tumors, overexpressed USP2a interacts with and stabilizes fatty acid synthase (FAS) (5, 8, 9), which is now recognized as a potentially therapeutic target in cancers of the breast, colon, endometrium, ovary, prostate, and thyroid (10–15). In LNCaP human prostate carcinoma cells, USP2a knockdown using targeted siRNA increases levels of poly-ubiquitinated FAS, reduces levels of FAS protein, and promotes induction of apoptosis (5, 7, 9).

The thiopurine analogues 6-mercaptopurine (6-MP) and 6-thioguanine (6-TG) have long been used in the treatment of acute lymphoblastic leukemia, the most common type of childhood cancer (16, 17). In addition to their anticancer effects, they have been used as clinically effective anti-inflammatory and immunosuppressive agents for over 50 years (18). Studies of 6-TG and 6-MP have revealed that although their therapeutic efficacies are similar, the two drugs differ in their mechanisms of action (16, 19, 20). The prodrug forms of 6-TG and 6-MP are enzymatically converted into cytotoxic nucleotides by hypoxanthine-guanine phosphoribosyl transferase. In addition, both drugs appear to have deubiquitinating and deISGylation activities, which enable them to inhibit coronavirus papain-like protease activity in cases of severe acute respiratory syndrome (21, 22). Thiopurines may thus belong to a new class of nonselective isopeptidase inhibitors with the ability to inhibit various isopeptidases and elicit accumulation of poly-ubiquitinated proteins (20). Recently, Dr. Chou’s laboratory demonstrated that a noncompetitive inhibition pattern best describes the inhibition of USP2a enzyme activity by 6-TG and 6-MP (23). This work also provided direct evidence of the functional impact of USP2a C276S mutation on its deubiquitinating activity.

Scientists are currently focusing on pharmacological disruption of DUB activity as a rationale for cancer therapy (24). In the present study, we sought to clarify the relationship between thiopurine analogs and USP2a, as both USP2a and USP14 will bind 6-MP and 6-TG (21, 22). We also examined the effects of 6-MP and 6-TG on USP2a target proteins to elucidate its functional roles. Our data suggest 6-MP and 6-TG may reduce the stability of some USP2a targets, including FAS and Mdm2, by inhibiting USP2a-catalyzed deubiquitination. These findings provide new insight into the anti-tumor functions of thiopurines.

## Materials and Methods

### TCGA-HNSC Data Mining

Gene expression profiles, clinical data and pathway activation scores in The Cancer Genome Atlas Head-Neck Squamous Cell Carcinoma (TCGA-HNSC) data collection were downloaded from UCSC XENA (https://xenabrowser.net/heatmap/). In the present study, we used gene expression, survival and pathway activity data. Briefly, patients in TCGA-HNSC were divided into a USP2 High group (30% from the patient with highest USP2 expression) and USP2 Low group (30% from the patient with lowest USP2 expression). For pathway activity, we downloaded “z scores of 1387 constituent PARADIGM pathways”, grouped based on USP2 expression and then analyzed for intergroup differences using GraphPAD (Version 9.1.2). Details of how these data were processed can be found on the UCSC Xena website.

### Cell Culture and Chemicals

A253 submaxillary carcinoma cells were cultured in McCoy’s 5a medium supplemented with 10% fetal bovine serum and 1% penicillin-streptomycin (Invitrogen, USA). The HeLa human cervical cancer cell line, DU-145 prostate cancer cell line, and Reh and SupB15 leukemia cell lines were cultured in Dulbecco’s modified Eagle’s medium supplemented with 10% phosphate buffered saline (PBS) and 1% penicillin-streptomycin (Invitrogen, USA). 6-MP and 6-TG were purchased from Sigma Aldrich (St. Louis, MO, USA).

### Western Blot Analysis

Cell lysates were prepared in lysis buffer (100 mM Tris-HCl [pH 8.0], 150 mM NaCl, 0.1% SDS, and 1% Triton X-100) at 4°C. Proteins in the cell extracts were separated by SDS-PAGE, transferred onto polyvinylidene difluoride membranes (Millipore, USA) and detected using antibodies against α-actinin (ACTN), ATF3, COX-2, cyclin D1, FAS, Mdm2, p21, p53, proliferating cell nuclear antigen (PCNA), USP2a (Santa Cruz Biotechnology, USA), Cdc2, cyclin B1, and p-Cdc2 (Cell Signaling, USA).

### Reverse Transcription-Polymerase Chain Reaction

Total RNA was isolated using TRIzol (Thermo Fisher Scientific) reagent according to the manufacturer’s instructions. One microgram of total RNA was subjected to reverse transcription using MMLV reverse transcriptase (Epicentre Biotechnologies, USA) for 60 min at 37°C. The PCR reactions were run on a Veriti Thermal Cycler (Applied Biosystems, USA). The following PCR primers were used for ATF3: forward primer 5’-GAGGATTTTGCTAACCTGAC-3’ and reverse primer 5’-TAGCTCTGCAATGTTCCTTC-3’; COX-2: forward primer 5’-TGGCGCTCAGCCATACAGCAA-3’ and reverse primer 5’-GGTGAAAGCTGGCCCTCGCT-3’; cyclin D1: forward primer 5’-ATGGAACACCAGCTCCTGTGCTGC-3’ and reverse primer 5’-TCAGATGTCCACGTCCCGCACGTCGG-3’; FAS: forward primer 5’-TGAGCCTCATGCGCCTGGAC-3’ and reverse primer 5’-CGCACCTCCTTGGCAAACAC-3’; GAPDH: forward primer 5’-CTTCATTGACCTCAACTAC-3’ and reverse primer 5’-GCCATCCACAGTCTTCTG-3’; Mdm2: forward primer 5’-CTTGATGCTGGTGTAAGTGA-3’ and reverse primer 5’-GTTGATGGCTGAGAATAGTC-3’; p21: forward primer 5’-CTGAGCCGCGACTGTGATGCG-3’ and reverse primer 5’-GGTCTGCCGCCGTTTTCGACC-3’; p53: forward primer 5’-GATGAAGCTCCCAGAATGCCAGAG-3’ and reverse primer 5’-GAGTTCCAAGGCCTCATTCAGCTC-3’; and USP2a: forward primer 5’-CGAGGTGAACCGAGTGACAC-3’ and reverse primer 5’-TGTTGTGAGCTTGCTGGTTCG-3’.

### Fluorescence-Activated Cell Sorting and Cell Cycle Profiling

The cell cycle distribution was determined by measuring DNA content using the FACS after staining with propidium iodide (PI). The cells were fixed in 70% ice-cold ethanol and kept at -20°C overnight. Before analysis, the harvested cells were washed twice with ice-cold PBS and stained with PI solution (5 μg/ml PI in PBS, 0.5% Triton X-100, and 0.5 μg/ml RNase A) for 30 min at 37°C in the dark. All the samples were analyzed using a FACSCalibur flow cytometer (BD Biosciences). Data were analyzed using Cell Quest Pro software (BD Biosciences).

### Plasmids and Transfection

*USP2a* wild-type expression vector was constructed by inserting the full-length PCR fragments into the pSG5.HA vectors *via* the *EcoR*I-*Xho*I restriction sites. A vector encoding a USP2a C267A mutant [pSG5.HA.USP2a (C267A)] was constructed using site-directed mutagenesis with a Promega Gene Edit kit (Promega, Madison, MI, USA). Cells plated in 6-well plates were transfected using jetPEI (PolyPlus-transfection, France) according to the manufacturer’s protocol; total DNA was adjusted to 1.0 µg by addition of the empty vector.

## Results

### Evaluating the Impact and Potential Mechanisms of USP2a Expression in HNSC Patients

**Figure 1:**
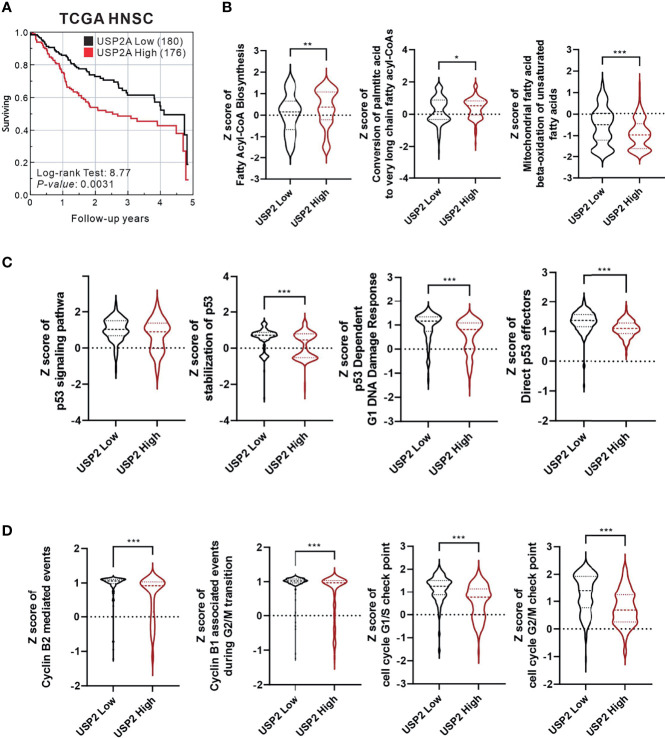
Analysis of The Cancer Genome Atlas Head-Neck Squamous Cell Carcinoma (TCGA-HNSC) data collection. The relationship between USP2a expression levels and **(A)** survival rate analyzed using the log-rank test; **(B)** fatty acid synthesis; **(C)** p53 function; **(D)** cell cycle progression. For all statistical analyses, unpaired t-tests were used, and results were considered significant at *p* < 0.05. *p < 0.05, **p < 0.01, and ***p < 0.001.

In biologically aggressive human tumors, overexpressed USP2a interacts with and stabilizes FAS, Mdm2, and cell cycle-related proteins (5–7). We first analyzed the relationship between the level of USP2a expression and survival using TCGA-HNSC data collection (**Figure 1A**). Our results indicated that the survival rate was lower among HNSC patients expressing higher levels of USP2a. The Z scores for fatty acyl-CoA biosynthesis and conversion of palmitic acid to very long chain fatty acyl-CoAs were higher in the USP2a High group, whereas the Z score of mitochondrial fatty acid beta-oxidation of unsaturated fatty acids was lower in the USP2a High group (**Figure 1B**). In the USP2a High group, we observed that Z scores for the p53 signaling pathway, stabilization of p53, p53-dependent G1 DNA damage response, direct p53 effectors (**Figure 1C**), cyclin B2-mediated events, cyclin B1-associated events during G2/M transition, the cell cycle G1/S check point, and cell cycle G2/M check point were all lower (**Figure 1D**).

### Multiple Pathways Used by 6-MP and 6-TG in the Regulation of USP2a Target Proteins in A253 Cells

**Figure 2:**
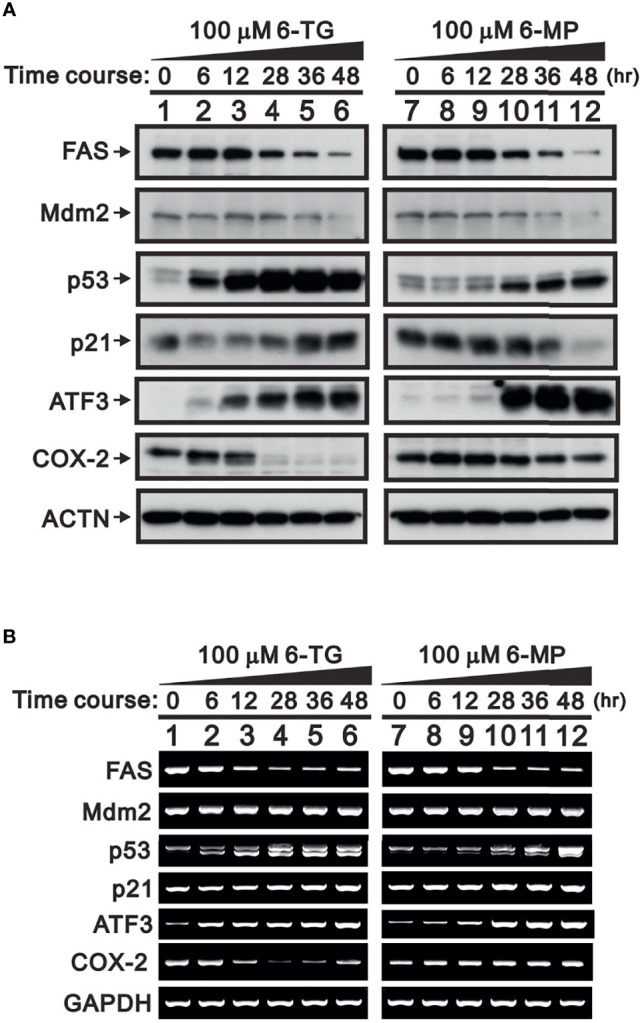
Time-course of the effects of 6-TG and 6-MP in A253 cells. Cells were treated for the indicated times with vehicle or 100 μM 6-TG or 6-MP, after which cell lysates were subject to **(A)** Western blotting analysis with antibodies against FAS, Mdm2, p53, p21, ATF3, and COX-2; or **(B)** RT-PCR analysis for FAS, Mdm2, p53, p21, ATF3, and COX‐2 mRNAs. ACTN is a protein loading control; GAPDH is an mRNA loading control.

FAS and Mdm2 are well-known target proteins for USP2a deubiquitinating activity (5, 25). Inhibition of USP2a could therefore result in their ubiquitin-dependent degradation. Consistent with that idea, in A253 cells treated with 100 μM 6-TG or 6-MP, Western blot analyses revealed time-dependent reductions in the levels of FAS and Mdm2 (**Figure 2A**). Moreover, the degradation of Mdm2 led to increases in the levels of p53 (26). The effects of 6-TG and 6-MP on levels of p21, a p53 target gene (27), were inconsistent, especially with 6-MP treatment (**Figure 2A**, compare lanes 7-12). RT-PCR analysis showed that neither 6-TG nor 6-MP affected Mdm2 mRNA expression, which is consistent them acting through USP2a inhibition. Both 6-TG and 6-MP suppressed expression of FAS mRNA (**Figure 2B**) while inducing expression of p53 mRNA and its splicing variant. On the other hand, they had no effect on expression of p21 mRNA.

A member of ATF/CREB transcriptional factor family, activating transcription factor 3 (ATF3) is a direct target of p53 that is rapidly induced by a wide range of cellular stresses (28, 29). Thus, increases in p53 elicited by treating A253 cells with 6-TG or 6-MP led to increases in both ATF3 mRNA and protein (**Figure 2**). A previous study demonstrated that ATF3 negatively regulates COX-2 during acute inflammation (30). We observed that the higher levels of ATF3 seen in 6-TG-treated A253 cells led to downregulation of COX-2 mRNA and protein; however, the effect of 6-MP on COX-2 expression was inconsistent (**Figures 2A, B**, compare lanes 7–12).

**Figure 3:**
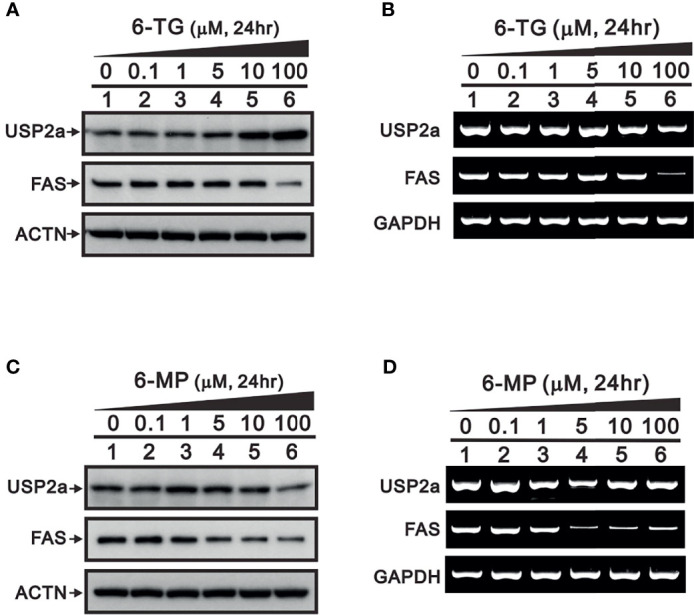
Dose-dependent effects of 6-TG and 6-MP on expression of USP2a protein and mRNA in A253 cells. Cells were treated for 24 h with the indicated concentration of 6-TG **(A, B)** or 6-MP **(C, D)**, after which cell lysates were subjected to **(A, C)** Western blotting analysis with antibodies against USP2a and FAS; or **(B, D)** RT‐PCR analysis for USP2a and FAS mRNAs. ACTN is a protein loading control; GAPDH is an mRNA loading control.

To confirm the importance of USP2a inhibition in FAS regulation by 6-TG and 6-MP, we examined their dose-dependent effects (**Figure 3**). We first observed that 6‐TG was able upregulate levels of USP2a protein while suppressing its mRNA expression (**Figures 3A, B**). Suppression of both USP2a mRNA and protein was observed with 6-MP (**Figures 3C, D**). Both 6-TG and 6-MP suppress levels of FAS protein, which is consistent with their dose-dependent suppression of FAS mRNA (**Figure 3**). These findings suggest that in A253 cells, 6-TG and 6-MP may modulate levels of USP2a target proteins, including FAS and Mdm2, through inhibition of USP2a’s deubiquitination activity or by directly suppressing mRNA expression of USP2a or its targets.

**Figure 4:**
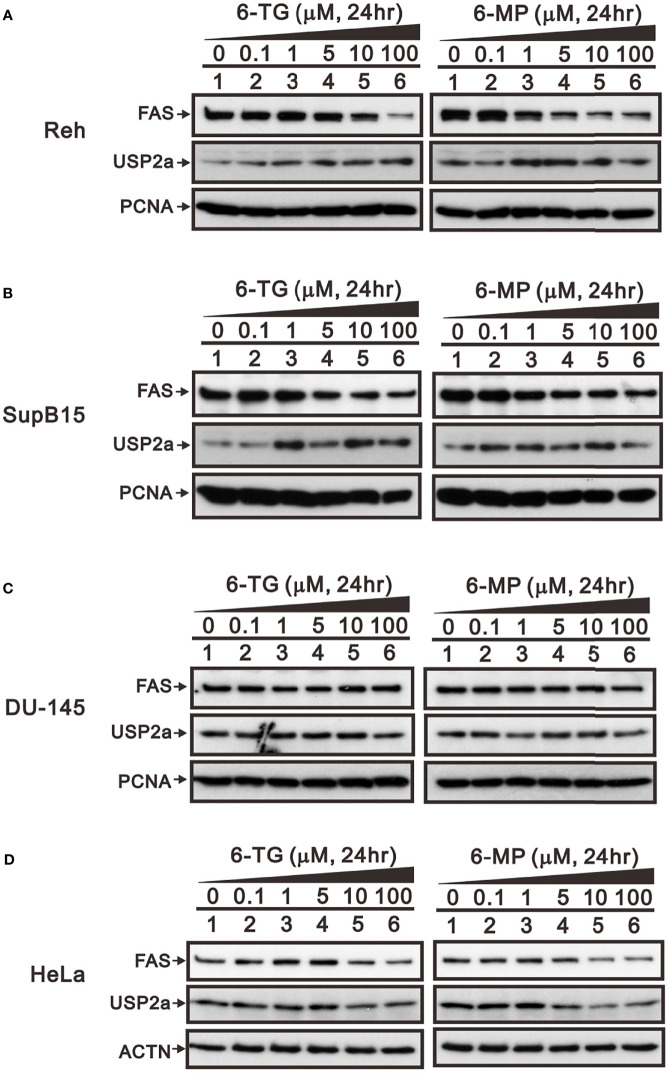
Effects of 6-TG and 6-MP on expression of FAS protein in several cell types. **(A)** Reh, **(B)** SupB15, **(C)** DU-145, and **(D)** HeLa cells were treated for 24 h with the indicated concentrations of 6‐TG or 6-MP, after which cell lysates were subjected to Western blotting analysis with antibodies against FAS and USP2a. PCNA or ACTN are protein loading controls.

To determine whether the observed suppression of FAS protein by 6-TG and 6‐MP in A253 cells was cell type-specific, we also tested their effects in the Reh and SupB15 leukemia cell lines, the DU-145 prostate cancer cell line, and the HeLa human cervical cancer cell line. Western blot analyses showed that 6-TG and 6-MP reduced the levels of FAS protein in Reh, SupB15, and HeLa cells (**Figures 4A, B, D**), but had no effect in DU-145 cells (**Figure 4C**). The effects of 6-TG and 6-MP on levels of USP2a protein varied among these four cell lines.

### 6-MP and 6-TG Regulate Cell-Cycle Progression-Related Proteins in A253 Cells

**Figure 5:**
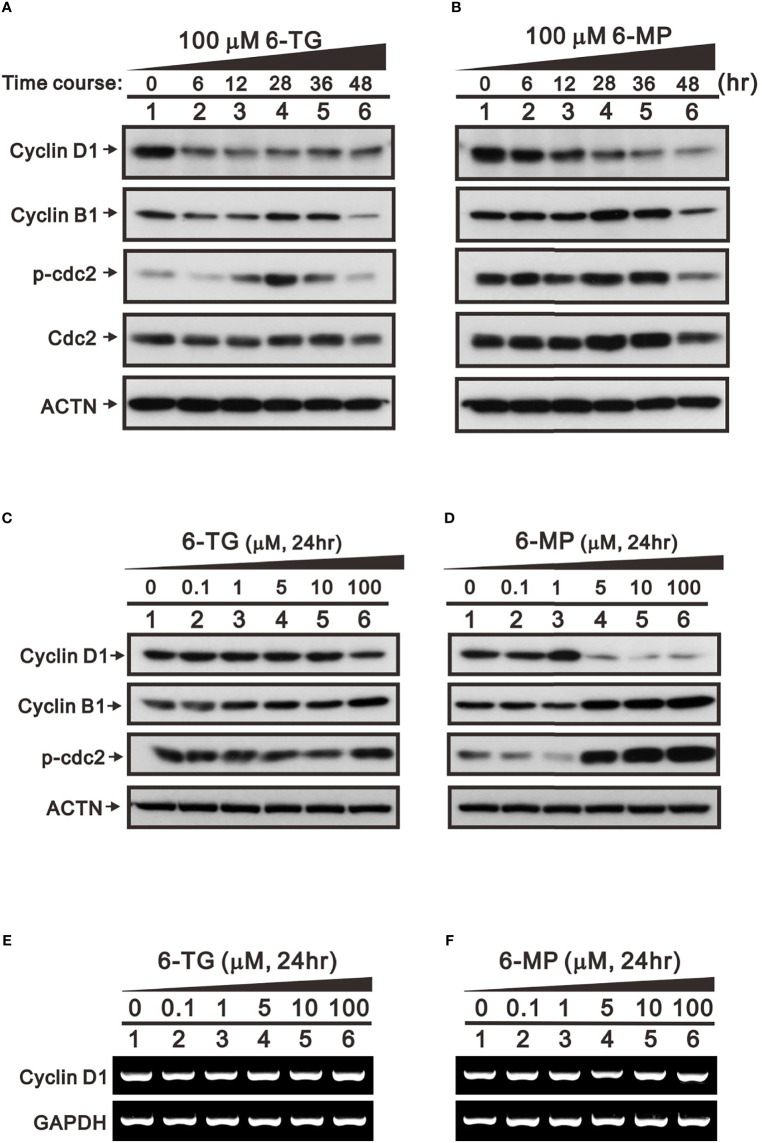
Time- and dose-dependent effects of 6-TG and 6-MP on expression of cell cycle-related proteins in A253 cells. Cells were treated for the indicated times **(A, B)** with 100 μM 6-TG or 6-MP or for 24 h **(C–F)** with the indicated concentration of 6-TG or 6‐MP. **(A–D)** Cell lysates were then subjected Western blotting analysis with antibodies against the indicated proteins. ACTN is a protein loading control. **(E, F)** Cell lysates were subjected to the RT-PCR analysis for cyclin D1 mRNA. GAPDH is an mRNA loading control.

**Figure 6:**
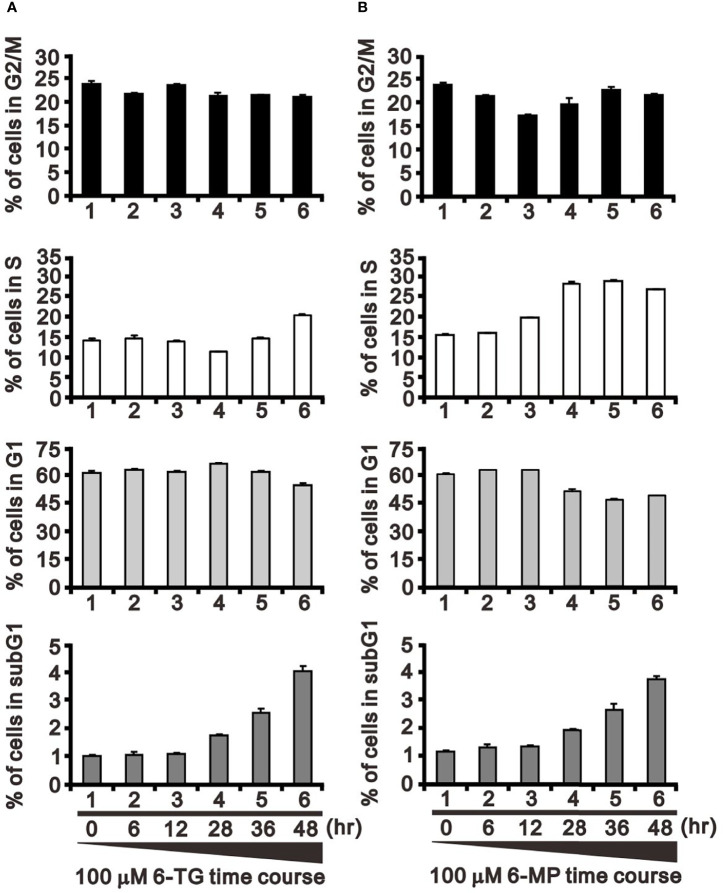
Effects of 6-TG and 6-MP on the cell cycle profile in A253 cells. Cells were treated for the indicated times with 100 μM 6-TG **(A)** or 6-MP **(B)**, after which the populations at the indicated cell cycle phases were quantified using flow cytometry. Dead cells were detected based on PI uptake.

In addition to FAS and Mdm2, cyclin D1 is a well-known target of USP2a deubiquitinating activity (31). Cyclin D1 plays a key role in G1 progression during the cell cycle. Our results indicate that 6-TG or 6-MP (100 μM) time-dependently suppressed levels of cyclin D1 protein (**Figures 5A, B**). The thiopurine-induced decline of cyclin D1 was accompanied by upregulation of ATF3. We also observed activation of the cdc2-cyclin B1 axis for transition from G2 to the mitotic phase in 6-TG- and 6‐MP-treated A253 cells. These effects of 6-TG and 6-MP on cyclin D1 and the cdc2‐cyclin B1 axis were dose-dependent (**Figures 5C, D**). The effects on cyclin D1 protein were not reflected by the corresponding mRNA levels (**Figures 5E, F**). This cell cycle profiling demonstrates that 6-TG and 6‐MP increase cell populations in subG1 and S phase by time-dependently decreasing the G1 phase population (**Figures 6A, B**).

### Exogenously Expressed USP2a Fails to Reverse the Effects of 6-TG and 6-MP in A253 Cells

**Figure 7:**
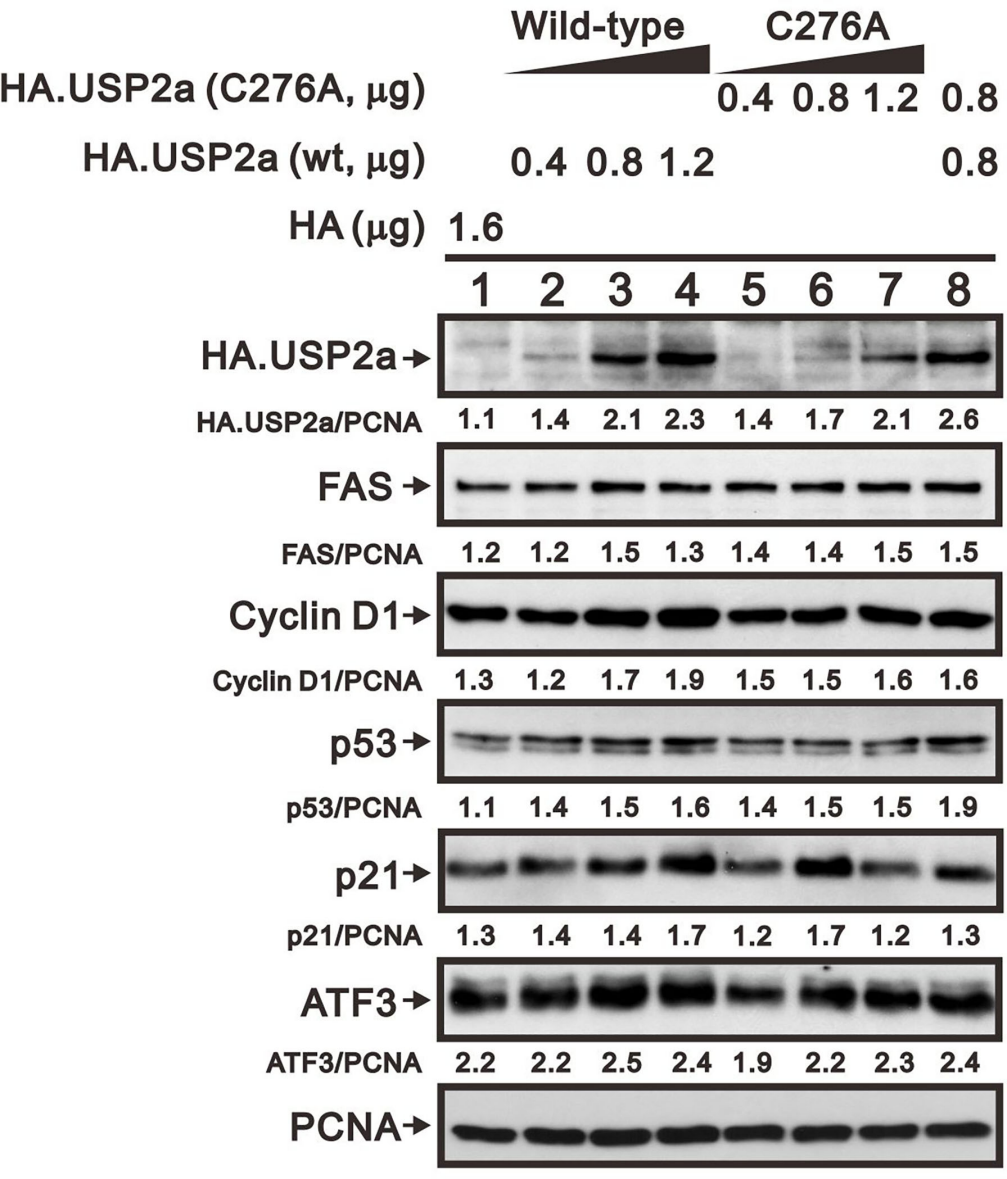
Levels of USP2a target proteins in A253 cells expressing wild-type USP2a or its C276A mutant. Cells were transiently transfected for 36 h with the indicated amount of pSG5.HA.USP2a (wild-type) or pSG5.HA.USP2a (C276A) vector, after which cell lysates were subject to Western blotting analysis with antibodies against HA, FAS, cyclin D1, p53, p21, and ATF3. PCNA is a loading control. Protein bands were quantified through pixel density scanning and evaluated using ImageJ software, version 1.44a (http://imagej.nih.gov/ij/).

**Figure 8:**
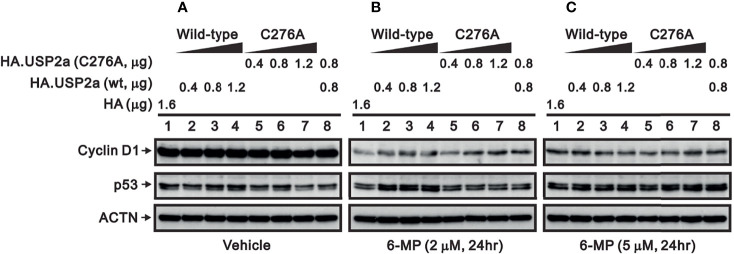
Effect of 6-MP on levels of USP2a target proteins in A253 cells expressing wild-type USP2a or its C276A mutant. Cells were transiently transfected for 16 h with the indicated amount of pSG5.HA.USP2a (wild-type) or pSG5.HA.USP2a (C276A) and then treated for 24 h with **(A)** vehicle, **(B)** 2 μM 6-MP, or **(C)** 5 μM 6-MP. Cell lysates were the subjected to Western blotting analysis with antibodies against cyclin D1 and p53. ACTN is a loading control.

The catalytic amino acid in the USP2a active site is Cys276, and substituting an Ala residue at that site eliminates the enzyme’s catalytic activity (23, 32). We tested whether the catalytic function of USP2a is involved in stabilizing target proteins using a USP2 C276A mutant (7). We observed that levels of both FAS and cyclin D1 proteins were dose-dependently increased in A253 cells transiently transfected with wild-type USP2a, but that increase was less evident in cells transfected with the USP2a C276A mutant (**Figure 7**). This suggests the deubiquitination activity of USP2a is important for stabilization of its target proteins, including FAS and cyclin D1. Similarly, we observed that transfection of wild-type USP2a dose-dependently increased levels of p53, p21, and ATF3 proteins (**Figure 7**). However, in cells treated with 6-MP, transfection of USP2a did not effectively inhibit the drug-induced cyclin D1 degradation (**Figure 8**, compare panels A and B or C), suggesting USP2a is not the only target of 6-MP. Induction of p53 protein was enhanced in USP2a transfectants treated with 6-MP (**Figure 8**, compare panels A and B or C).

## Discussion

Our results demonstrate that levels of FAS, cyclin D1, and Mdm2 proteins are reduced in cells treated with 6-TG or 6-MP. They further suggest this effect of 6-TG and 6-MP reflects their ability to inhibit USP2a and, thus, increase ubiquitination and proteasomal degradation. By inhibiting ubiquitination, DUBs play a crucial role in determining the cellular fate of numerous proteins (2). USP2a is member of the DUB family and may function in the removal of ubiquitin from specific targets to prevent their degradation (3). In addition to the reported discovery of two small-molecule inhibitors of USP2a (33, 34), the noncompetitive inhibition of USP2a enzyme activity by 6-TG was recently reported, and the kinetic and catalytic mechanism was confirmed by X-ray crystallography (23). In the present study, we examined whether 6‐TG or 6-MP could serve as an effective USP2a inhibitor in cells. Our findings suggest that 6‐TG or 6-MP could serve as an effective USP2a inhibitor in cells and stabilize USP2a’s target proteins, including FAS, Mdm2, and cyclin D1. However, the inability of exogenous USP2a to offset the inhibitory effect of 6-TG and 6-MP on USP2a activity suggests 6-TG and 6-MP exert other effects that predominate in A253 cells. In addition to inhibiting USP2a enzyme activity, our data suggest 6-TG and 6-MP may directly regulate USP2a mRNA and protein expression, though we detected differences in the cellular responses to the two drugs. This may reflect, in part, a difference in the susceptibility of 6-TG and 6‐MP to S-methylation catalyzed by thiopurine methyltransferase, an enzyme involved in their metabolism (19). However, the detailed mechanisms of 6-TG and 6-MP remain to be investigated in the future.

Proteins known to be targets of USP2a include FAS, CRY1, cyclin A1, cyclin D1, EGFR, Mdm2, Aurora-A, and RIP kinase 1 (5, 25, 31, 32, 35–38). The impact of USP2a activity will depend on the function its target proteins and the effect of their stabilization on the activities of relevant signaling networks. FAS is often overexpressed in aggressive human tumors, including prostate cancer and glioma. In addition, p53 is not a direct target of USP2a, but Mdm2 is a target. Destabilization of Mdm2 decreases the degradation of p53 and, in turn, leads to induction of p53 target genes, including stress proteins such as p21 and ATF3. The Mdm2-p53-ATF3-COX-2 axis provides a case in which an indirect effect of USP2a may play an important role mediated through p53, ATF3, or COX-2 protein. Thus, the indirect effects of 6-TG and 6-MP may open new avenues in the treatment of various cancers. In that context, although the use of 6-TG and 6-MP in the treatment of leukemia is well established, their modes of action remain controversial (39). The combined direct and indirect effects of these thiopurine drugs, which likely involve protein-protein interactions that are not well defined, may underlie the controversial findings from the present working models.

6-TG and 6-MP are well-studied thiopurine analogs that have both anticancer and immunosuppressive activities (18, 40). All thiopurines are prodrugs, and their cytotoxic activities are regulated by endogenous enzymes in different metabolic pathways (16, 40). In general, the cytotoxicity of 6-TG is believed to mainly reflect incorporation of 6-thioguanine nucleotides into DNA, whereas 6-MP exerts its effects mainly through inhibition of purine biosynthesis. The observation that thiopurine analogues inhibit coronavirus papain-like protease in severe acute respiratory syndrome, prompted us to test whether 6-TG and 6-MP would act as inhibitors of USP2a. We observed that 6-TG and 6-MP differentially inhibit the deubiquitination activity of USP2a and modulate the mRNA and protein expression of USP2a and FAS. Although multiple working mechanisms for antitumor functions of 6-TG and 6-MP have been reported (18, 41, 42), the clinical responses suggest that combining thiopurines with a natural compound or other agent, such as methylthioadenosine or methotrexate, might enhance therapeutic efficacy in methylthioadenosine phosphorylase-deficient tumors (16).

FAS is present at high levels in many human cancers, including colon, endometrial, ovarian, prostate, and thyroid cancer (10–15). FAS is well-known to catalyze the NADPH-dependent condensation of malonyl-CoA and acetyl-CoA to produce the 16-carbon saturated free fatty acid palmitate. The association of FAS expression with tumor virulence suggests FAS activity is vital to human cancer cells. Several studies have shown that FAS inhibition using siRNAs or small-molecule inhibitors induces tumor cell apoptosis (43–45). By inhibiting USP2a, 6-TG and 6-MP destabilize FAS. Treatment with 6-TG and 6-MP also leads to increases in the population of cells in subG1 phase, which may be related to the induction of apoptosis mediated through FAS degradation. In addition to inducing apoptosis, the effects of thiopurines on FAS have other profound and complicated implications for the synthesis of nucleotide analogs in cancer cells. Those interesting issues will be addressed in the future.

## Conclusions

In summary, our findings verify the impact 6-TG- and 6-MP-mediated inhibition of USP2a on its target proteins, including FAS, Mdm2, and cyclin D1. 6-TG and 6-MP also suppressed levels of both USP2a mRNA and protein. The effects of 6-TG and 6‐MP were not cell type-specific, as they elicited similar decreases in FAS protein in leukemia, prostate and cervical cancer cell lines. 6-TG and 6-MP had effects on several cell cycle proteins, including another USP2a target protein, cyclin D1. The cell populations in subG1 or S phase were increased by 6-TG and 6-MP, which was accompanied by reductions in G1 phase cells. Notably, exogenous overexpression of USP2a failed to offset the effects of 6-TG and 6-MP on levels of USP2a target proteins, suggesting 6-TG and 6-MP do not act solely through inhibition of USP2a. Examination of the interplay among cancer biology, metabolism, and small molecule drug may open avenues to devise new diagnostic and treatment strategies for cancer patients.

## Data Availability Statement

The original contributions presented in the study are included in the article/supplementary material. Further inquiries can be directed to the corresponding author.

## Author Contributions

C-PC conceived, analyzed data, and wrote the paper. S-TL and Y-LC carried out experiments and analyzed data. S-MH and C-LH conceived the study, participated in its design, and helped draft the manuscript. All authors contributed to the article and approved the submitted version.

## Funding

This work was supported by grants from the Ministry of National Defense-Medical Affairs Bureau [MND-MAB–109–084 and MND-MAB–110–089 to S-MH], the Ministry of Science and Technology [MOST 105-2314–B–016–047, 106-2314–B–016–039, and 110-2314-B-016-056 to C-LH], Taiwan, ROC.

## Conflict of Interest

The authors declare that the research was conducted in the absence of any commercial or financial relationships that could be construed as a potential conflict of interest.

## Publisher’s Note

All claims expressed in this article are solely those of the authors and do not necessarily represent those of their affiliated organizations, or those of the publisher, the editors and the reviewers. Any product that may be evaluated in this article, or claim that may be made by its manufacturer, is not guaranteed or endorsed by the publisher.

## Abbreviations

6-MP, 6-mercaptopurine; 6-TG, 6-thioguanine; ACTN, α-actinin; DMEM, Dulbecco’s modified Eagle’s medium; DUBs, deubiquitinases; FAS, fatty acid synthase; FACS, fluorescence-activated cell sorting; FBS, fetal bovine serum; PCNA, proliferating cell nuclear antigen; PARP, poly-ADP-ribose polymerase; PBS, phosphate buffered saline; PI, propidium iodide; RT-PCR, reverse transcription-polymerase chain reaction; USPs, Ubiquitin-specific proteases.
